# Towards Enhanced Battery Thermal Safety: A Lightweight and Mechanically Robust Aerogel with Superior Insulation

**DOI:** 10.3390/gels12010054

**Published:** 2026-01-05

**Authors:** Yin Chen, Ruinan Sheng, Mingyi Chen

**Affiliations:** School of the Environment and Safety Engineering, Jiangsu University, Zhenjiang 212013, China; chenyin@ujs.edu.cn (Y.C.); 2222209099@stmail.ujs.edu.cn (R.S.)

**Keywords:** lithium-ion battery, thermal safety, thermal runaway propagation, aerogel

## Abstract

With the continuous increase in energy density of lithium-ion batteries, thermal safety has become a critical constraint on their further development. To address the limitations of mechanical brittleness and high-temperature infrared transparency in SiO_2_ aerogels for thermal safety applications in lithium-ion batteries, this study developed a novel nanofiber aerogel composite by incorporating chitosan and MXene into a SiO_2_ aerogel matrix. This material retains the characteristics of being ultra-lightweight and highly elastic while significantly enhancing mechanical strength and high-temperature insulation performance. It exhibits a thermal conductivity of 0.034 W/m K at room temperature and 0.053 W/m K at 400 °C, alongside a compressive strength of 1.172 MPa. In battery thermal runaway propagation tests, the aerogel successfully prevented propagation in serially connected and electrically isolated systems, and delayed thermal runaway propagation by 35 s in a parallel system, demonstrating excellent thermal runaway suppression capability. This work provides an effective material solution for the practical application of high-performance thermal insulation aerogels in battery safety protection and offers inspiration for developing new insulating ceramic aerogels.

## 1. Introduction

Aerogels, as nanoporous materials with high porosity, extremely low thermal conductivity, and ultra-low density, show broad application prospects in thermal management, energy storage, and environmental protection [1]. Especially with the rapid development of electric vehicles and large-scale energy storage technologies, thermal safety management of lithium-ion batteries has become a key factor restricting their safety performance and cycle life [2,3]. In battery modules, effective thermal barriers can significantly delay or even prevent the propagation of thermal runaway, avoiding catastrophic thermal events [4,5].

In recent years, biomass polymer-based composite aerogels have attracted widespread attention due to their sustainability, abundant functional groups, and tunable microstructure [6,7]. A fully biomass-based aerogel fabricated from ammonium alginate and phytic acid simultaneously achieves ultrahigh mechanical strength, excellent flame retardancy, low thermal conductivity, and high biodegradability at an extremely low density [8]. Among them, chitosan, as the second most abundant natural polysaccharide, possesses abundant amino and hydroxyl sites in its molecular chain, providing excellent chemical reactivity and cross-linking ability, making it an ideal substrate for constructing high-performance composite aerogels [9]. A lightweight chitosan-derived carbon aerogel incorporating electrostatically assembled BPNS@MOF achieves exceptional microwave absorption performance while simultaneously offering ultralow thermal conductivity, excellent fire resistance, and outstanding sound absorption, demonstrating strong potential for multifunctional aerospace and defense applications [10]. Meanwhile, MXene [11], as an emerging class of two-dimensional inorganic nanomaterials, shows great potential in enhancing the mechanical and thermal insulation properties of polymer matrices due to its unique layered structure, high specific surface area, and excellent infrared shielding properties [12,13]. Previous studies have confirmed that MXene materials possess extremely low intrinsic emissivity across a wide infrared band and a high extinction coefficient for near-infrared light [14,15]. A hyperelastic, air-stable MXene–cellulose aerogel encapsulated with fire-retardant thermoplastic polyurethane achieves record-high electromagnetic interference shielding effectiveness and MXene utilization efficiency at ultralow MXene loading, while simultaneously offering excellent mechanical durability, stability, and fire safety for multifunctional applications [16]. Despite the significant progress made in the above research, the application of aerogel insulation materials containing chitosan and MXene to the thermal safety protection of high-energy-density lithium-ion battery modules still faces unique challenges from the application scenarios.

On the other hand, various innovative aerogel design strategies have been proposed to address the thermal insulation challenges in extreme environments [17,18]. Gao’s team developed an innovative two-dimensional channel-confined chemistry strategy to construct ultralight aerogels with a “dome-cell” structure. These aerogels exhibit excellent elasticity and thermal stability over an ultrawide temperature range of 4.2 K to 2273 K [19]. They further elucidated the mechanical advantages of this type of dome-structured aerogel, pointing out that its hyperbolic configuration can generate a high-density, uniformly distributed fold network under compressive loading, effectively storing elastic strain energy and thus maintaining structural integrity under extreme thermo-mechanical coupling environments. In the field of biomimetic design, Zhuo et al., inspired by leaf structures, successfully synthesized a silica/chitosan/zirconia fiber composite aerogel (SCZ). By mimicking the epidermis, mesophyll, and vein structures of a leaf, they achieved a balance between low density, high mechanical strength, and excellent high-temperature dimensional stability [20]. Bai et al. developed a Si@ANF/CNF porous aerogel with negative Poisson qubit properties, exhibiting thermal conductivity of only 0.037 W/m·K at 80 °C and 0.044 W/m·K at 160 °C [21]. Amir Varamesh et al. used a layer-by-layer self-assembly technology to deposit bio-based flame retardants. The aerogels exhibited excellent flame retardancy, self-extinguishing ability, and good thermal insulation properties, providing an environmentally friendly alternative [22]. In the field of battery thermal management, Sui et al. developed an adaptive heat dissipation film mimicking mammalian skin sweat glands, capable of reducing battery temperature through evaporative cooling while providing flame-retardant protection [23]. Meanwhile, Wang et al. designed an inorganic phase change film with a dual thermal management mechanism of “thermal conduction + thermal absorption” using composite phase change energy storage materials, effectively buffering the instantaneous heat flux of electronic devices [24]. Liu et al. proposed a multi-network gel inspired by mobile power supply structures, combined with flexible composite phase change materials, to enhance the thermal protection of lithium-ion battery modules [25]. Jiang et al. prepared aerogel composite felts using the sol–gel method and supercritical drying, which have low thermal conductivity and good mechanical properties. Simulation and experimental verification showed that they can effectively delay the propagation of thermal runaway [26]. Cai et al. reported a smart thermal management fireproof composite aerogel with dynamic self-switching daytime radiative cooling and photothermal conversion capabilities, for safer, year-round energy saving [17]. Chen et al. [27] designed a sandwich structure composed of a flame-retardant phase change material and an aerogel felt to suppress the propagation of thermal runaway in lithium-ion battery modules. This work, by combining the thermal management properties of the flame-retardant phase change material with the thermal insulation properties of the aerogel felt, provides a valuable reference for efficiently suppressing and blocking thermal runaway. Although some progress has been made in recent years in suppressing the propagation of thermal runaway in battery modules, existing research still falls short of fully covering the propagation of multi-scale coupling under complex operating conditions [5]. A large amount of systematic research remains to be conducted on control strategies focusing on novel suppression materials and optimized structural design.

Despite significant progress in aerogel material research, traditional aerogels still have significant shortcomings in battery thermal runaway protection applications [28,29]. Firstly, most studies focus on optimizing the thermal insulation or mechanical properties of aerogels individually, while material systems that can simultaneously meet the thermal insulation and mechanical strength requirements under extreme environments remain limited. Especially under high-temperature conditions, it is often difficult to simultaneously achieve both structural stability and elastic recovery capability in aerogels. Secondly, the effectiveness of existing aerogel materials in suppressing battery thermal runaway propagation has not been systematically evaluated. Studies have shown that excessively superior thermal insulation performance may lead to heat accumulation, which in turn exacerbates the total heat release during thermal runaway [30]. How to effectively block heat propagation while avoiding excessive heat accumulation is a key challenge in the design of current aerogel insulation materials. Furthermore, although biomass-based aerogels are environmentally friendly and easily functionalized, their thermal stability and flame retardancy at high temperatures are generally inferior to those of all-inorganic aerogels. How to achieve high-temperature stability and high-efficiency flame retardancy of biomass-based aerogels through reasonable component design and structural control is an important issue for promoting their practical application. It is also need to design a material with a comprehensive performance balance under the complex environment of thermo-mechanical-electric coupling, specifically for the medium-to-high temperature application scenario (~400–800 °C) of thermal safety protection for lithium-ion batteries.

Addressing the research gaps, this study proposes a novel SiO_2_/chitosan/MXene (SCM) composite aerogel mat featuring a multi-component synergistic design and bio-inspired heterogeneous structure, based on the goal of effectively blocking the propagation of thermal runaway and maintaining structural stability in battery modules. By incorporating MXene as an infrared opacifier to reduce radiative thermal conductivity and chitosan as a biopolymer binder to enhance mechanical strength, a stable ternary network is constructed through hydrogen bonding between surface functional groups. The resulting aerogel demonstrates ultra-low thermal conductivity, improved compressive strength, and effectively suppresses thermal runaway propagation in series and non-connected battery systems, offering a balanced material solution for high-safety battery thermal management. It validates the application potential in battery thermal management, providing theoretical and experimental basis for the development of next-generation high-performance battery thermal insulation materials.

## 2. Results and Discussion

Multilayer MXene nanosheets were obtained by etching the Ti_3_AlC_2_ (MAX) material with an HCl/LiF solution to remove the Al layer. Single-/few-layer MXene nanosheets were subsequently achieved through further ultrasonication and centrifugation. MXene nanosheets are negatively charged and hydrophilic due to the presence of abundant terminal functional groups (-OH, =O, and -F). Consequently, they can form stable suspensions in water, facilitated by electrostatic repulsion between adjacent MXene nanosheets. The prepared chitosan solution was then mixed with the MXene aqueous solution in a specific ratio, followed by ultrasonication. The mixed solution could be cross-linked by adding glutaraldehyde to form a hydrogel. A Schiff base reaction occurs between GA molecules and CS chains. Additionally, electrostatic repulsion between the negatively charged CS and MXene ensures the excellent dispersion of MXene nanosheets within the CS hydrogel. MXene sheets contain elements such as H, O, or F, providing abundant functional groups like -OH and -NH_2_. After 8 h of reaction, the TEOS solution was added. As the reaction proceeded, the in situ formed SiO_2_ became randomly mixed with MXene. Following the reaction between silica and chitosan, the aggregated silica structures became entangled with chitosan at the molecular level, generating a three-dimensional semi-interpenetrating network and forming cluster-like structures. Meanwhile, the hydroxyl functional groups on the surface of the silica clusters formed hydrogen bonds with the oxygen-containing functional groups on the MXene surface. These bonds act as pillars attached between the monolayer MXene sheets, supporting them to form a three-dimensional space. The molecular structure of chitosan is characterized by its rich polar hydroxyl groups, which enhance the strength of intermolecular hydrogen bonding. Finally, the SCM composite nanofiber aerogel was obtained after freeze-drying.

**Figure 1:**
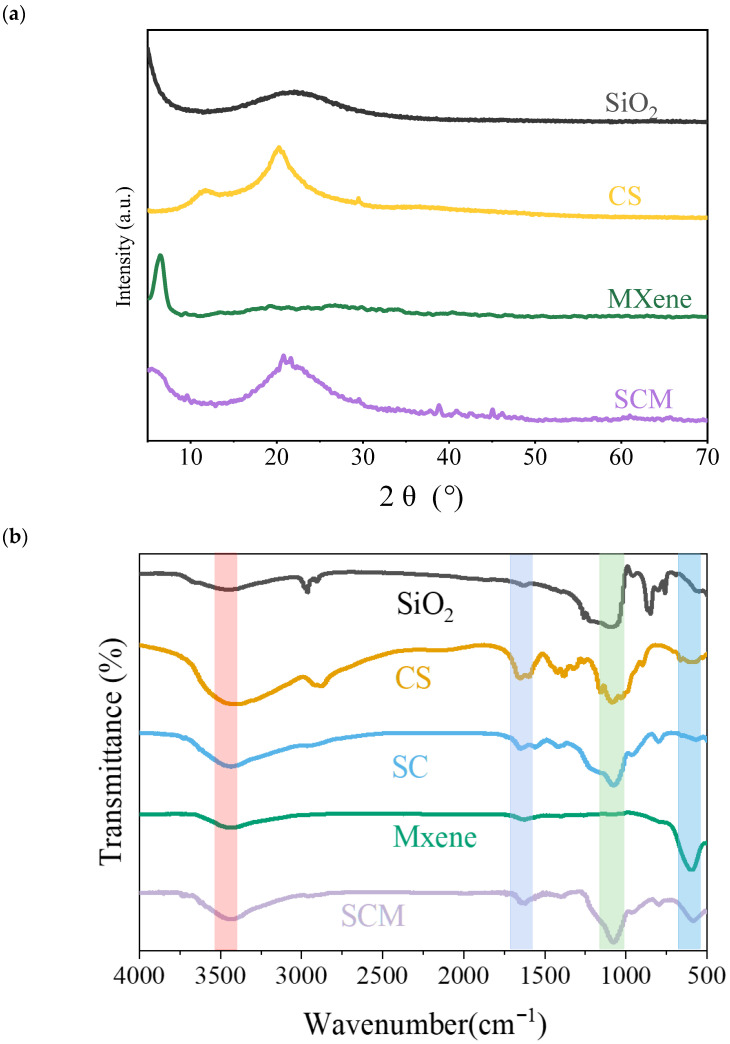
(**a**) Composite aerogel XRD spectra; (**b**) FT-IR image of composite aerogel.

The synthesized composite nanofiber aerogel underwent a series of tests and characterizations. The XRD spectrum of the composite aerogel is shown in Figure 1a. The XRD pattern of the as-synthesized SCM shows very few significant peaks. The CS matrix covered the surface of the MXene nanosheets, which may reduce the crystallinity of MXene, making its characteristic peaks less pronounced. The typical characteristic peak of MXene nanosheets at 2θ = 6.9° either disappeared or broadened. Due to the successful introduction of amorphous SiO_2_ into the SCM samples, the interlayer spacing of the material increased, and a new broad peak appeared at approximately 23°. The FTIR spectrum of the composite aerogel is shown in Figure 1b. Characteristic peaks of SCM at 547 cm^−1^ and 1090 cm^−1^ can be clearly observed in the spectrum, attributed to the bending vibration of Ti-O in MXene sheets and the stretching vibration of C-O, respectively, indicating the reaction between SiO_2_ and CS to form siloxane bonds. Furthermore, the peak near 1520 cm^−1^ corresponds to the in-plane and out-of-plane stretching vibrations of carboxyl groups. The broad absorption band for -OH groups around 3433 cm^−1^ in SCM decreased, which is attributed to the hydrogen bonding interactions among the three components.

**Figure 2:**
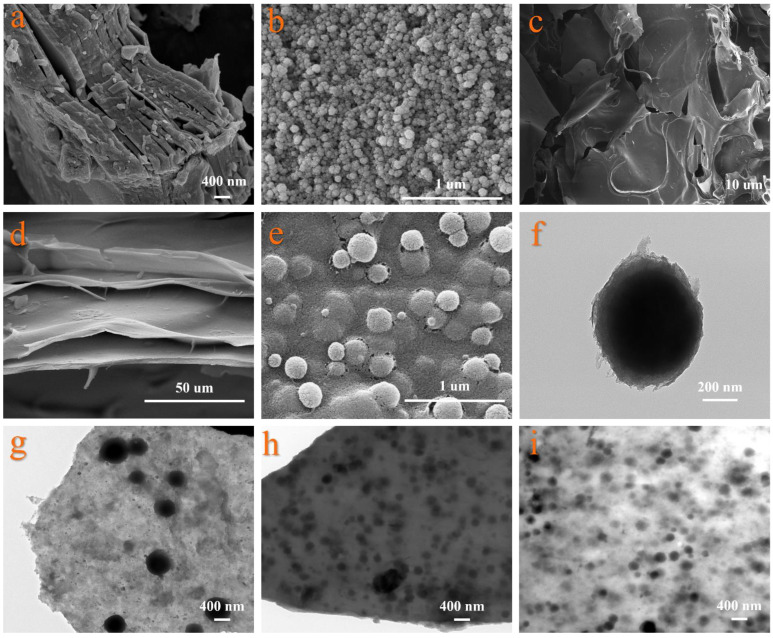
SEM and TEM images of cross-section and surface of composite aerogel mat matrix; (**a**) SEM image of MXene sheet; (**b**) Scanning electron microscopy image of SiO_2_ aerogel; (**c**) Scanning electron microscopy image of chitosan aerogel; (**d**) SCM-1 cross-section scanning electron microscope image; (**e**) SEM image of the SCM-1 surface; (**f**) Partial enlargement of the SEM image of the SCM-1 surface; (**g**) Transmission electron microscopy image of SCM-1; (**h**) Transmission electron microscopy of SCM-2; (**i**) Transmission electron microscopy of SCM-3.

Figure 2a–i presents the SEM and TEM micrographs of the composite aerogels. The images reveal a networked and bridged structure with various annular pores formed by the sublimation of ice crystals. Figure 2a displays the SEM image of MXene sheets, where multilayered MXene is closely stacked, forming a distinct accordion-like structure. Figure 2b shows the SEM image of pure silica aerogel, revealing that nano-SiO_2_ particles aggregate into clustered structures. Figure 2c is the SEM image of pure chitosan (CS) aerogel, which exhibits a porous three-dimensional network structure overall. Figure 2d indicates that the tightly stacked MXene sheets were successfully intercalated by silica spheres, resulting in an accordion-like structure with increased interlayer spacing, which provides effective support for the MXene nanosheets. The pores of SCM-1 aerogel felt are mainly distributed in the range of 10–20 μm. Figure 2e illustrates that the clustered silica spheres react with the oxygen-containing functional groups on the MXene surface to form hydrogen bonds, which act as pillars attached between the monolayer MXene sheets. Figure 2f shows that after the silica sol reacts with the chitosan solution, the CS partially hydrolyzes. The primary SiO_2_ particles are stabilized and coated by the chitosan biopolymer, leading to the formation of larger and denser secondary silica particles. These secondary particles are effectively glued together by chitosan, further promoting the formation of cluster structures. These silicon clusters have an average diameter of approximately 400 nm. The numerous pores between the clusters, effectively limiting convective heat transfer of air molecules and increasing the thermal resistance of gas conduction. This is the structural basis for the low thermal conductivity of SCM at room temperature. The abundant H, O, and F elements in the MXene sheets facilitate the formation of hydrogen bonds among MXene, CS, and SiO_2_. The hydroxyl and amino functional groups in chitosan molecules undergo self-crosslinking, while the amino groups also participate in acetal reactions with glutaraldehyde. These interactions collectively reinforce the three-dimensional network structure and connect the MXene sheets together, resulting in an aerogel with enhanced mechanical strength. The TEM images of SCM-1, SCM-2, and SCM-3 are shown in Figure 2g–i. As the content of silica sol in the composition increases, the silica spheres become distributed more densely on the MXene basal planes. This uniform dispersion ensures that at high temperatures, infrared photons are frequently scattered or absorbed by the MXene sheets along their transmission path, thereby suppressing the contribution of radiative heat transfer. Consequently, under high-temperature conditions, compared to SCM-2 and SCM-3, SCM-1 exhibits lower infrared transmittance, a smaller thermal conductivity, and a slower surface temperature rise rate.

**Figure 3:**
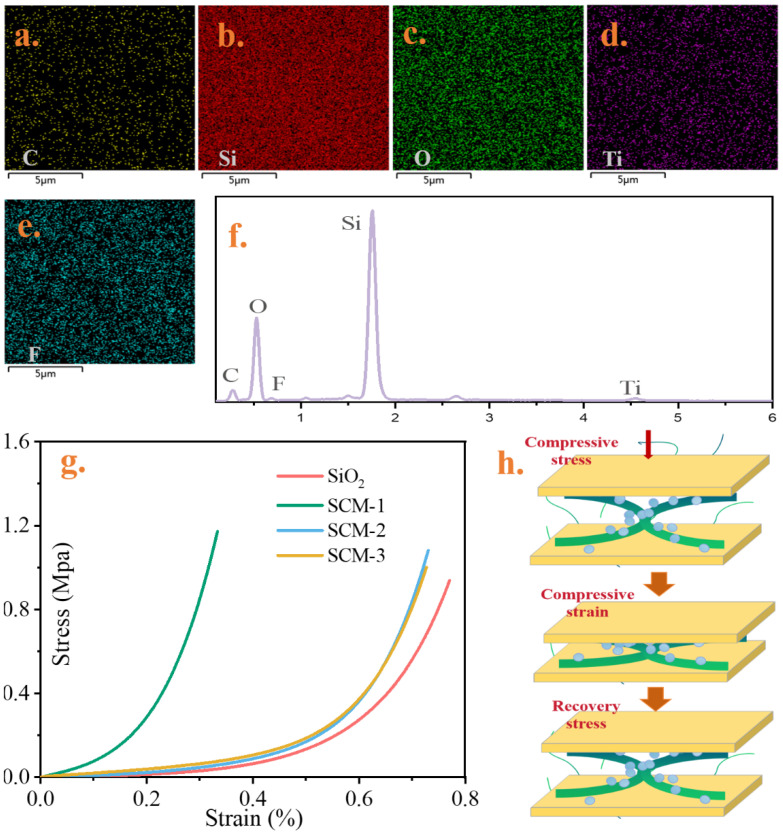
TEM images of composite aerogel materials, (**a**–**e**) are the distribution of C, Si, O, Ti and F, respectively, and (**f**) the element content images. (**g**) the stress–strain curve; (**h**) schematic diagram of compression-recovery of a single nanofiber unit.

Figure 3a–e shows the elemental distribution maps of the cross-section and surface of the high-temperature insulation and flame-retardant aerogel felt matrix. The images indicate that elements such as C, Si, O, F, and Ti are uniformly distributed on the aerogel surface. It can also be proved from element content images in Figure 3f. Among them, Si and O exhibit the strongest signals, indicating that the material is dominated by a silicon–oxygen framework. Although the signal intensity of F is relatively weak, the enlarged low-energy spectrum clearly demonstrates its existence, suggesting the successful introduction of fluorine species. Ti form MXene is also detected with a minor signal, implying its incorporation as a secondary component. As can be seen from the compressive stress–strain curves in Figure 3g, the aerogel felt after sol–gel processing exhibits ductile material behavior. Upon initial loading, a significant strain hysteresis is observed. Compared to the SiO_2_ aerogel felt, samples with higher MXene content demonstrate stronger compressive resistance. This enhancement is attributed to the effective interfacial reinforcement provided by CS macromolecular chains. The primary colloidal silica particles are stabilized/coated by the chitosan biopolymer, which further regulates the structural formation of secondary particles/clusters. The chemical interaction between silica and chitosan is likely mediated at the particle surface by a siloxane adsorption layer on the biopolymer. During gelation, the network of secondary particle morphologies is effectively “glued” together by chitosan. Hydrogen bonding interactions between the chitosan hydrogel and MXene nanosheets result in the MXene nanosheets being tightly encapsulated by a carbon matrix, forming a nanostructured framework as illustrated in Figure 3h. The absolute compressive strength of pure SiO_2_ aerogel felt was measured to be 0.92 MPa. Therefore, the compressive strength of the SCM-1 composite material was significantly improved by 27%. To assess the practical application value of this mechanical property, it is instructive to compare it with typical stresses in battery modules. The compressive strength of SCM-1 (1.172 MPa) is within the pressure range of normal battery operation, and its inherent strength is sufficient to meet the requirements of the application environment. Crucially, the main mechanical advantage of MXene-chitosan reinforced materials lies not only in the higher peak load capacity but also in the structural cohesion they impart, which prevents the aerogel from fracturing or shedding particles under load. This integrity is essential for ensuring the long-term reliability of the insulation layer, maintaining electrical isolation between cells, and enabling the material to function effectively as a cell spacer in constrained components.

**Figure 4:**
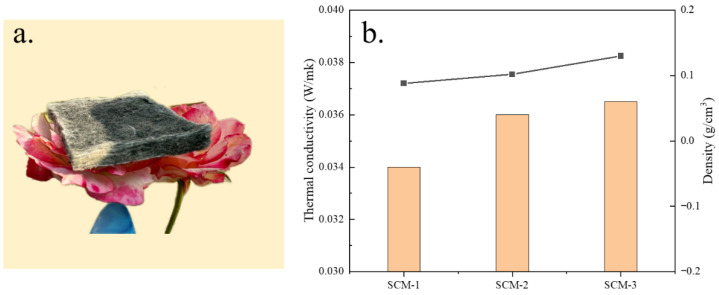
(**a**) Composite aerogel felt placed on stamens, (**b**) Thermal conductivity and density of composite aerogel felt with different shade contents.

Lightweight thermal insulation materials play a significant role in improving thermal efficiency and conserving energy. In addition to excellent compressive resistance, nanofiber aerogels are recognized as efficient thermal insulation materials due to their extremely low thermal expansion and density. As shown in Figure 4a, the as-prepared composite aerogel felt can stably stand on a flower, demonstrating its ultra-lightweight characteristic. The thermal conductivity of the aerogels was tested using the transient hot-wire method. Measurements were performed using the transient planar heat source method with a Xiangtan Dra-Ⅲ multifunctional thermal conductivity meter. The probe was a standard insulated planar sensor made of nickel wire with a diameter of Φ15 mm. The SCM composite aerogel felt samples used for testing were cut into cubes, and stacked to ensure a thickness of not less than 10 mm. Each sample ratio was measured at least three times independently at 25 °C (room temperature), and the final reported value was its arithmetic mean. Measurement relative error: ≤3%, repeatability error: ≤3%. Figure 4b shows the thermal conductivity and density of the nanofiber aerogels with different MXene aerogel contents. As the MXene content increases, the thermal conductivity of the nanofiber aerogels decreases significantly. The room-temperature thermal conductivity of SCM-1 is only 0.034 W/(m·K). The densities of all samples are less than 0.13 g/cm^3^ and decrease with increasing MXene aerogel content. This may be attributed to the introduction of MXene nanosheets effectively reducing the linear shrinkage of the composite aerogel, thereby lowering its density.

**Figure 5:**
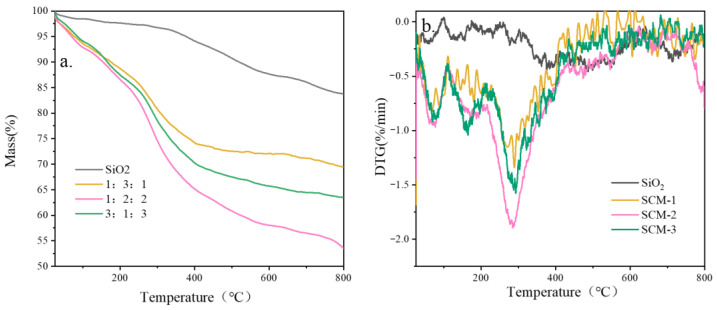
TG-DTG curves of composite aerogel mats with different components: (**a**) weight loss, (**b**) DTG.

Figure 5 shows the thermogravimetric (TG) curves of the high-temperature insulation and flame-retardant aerogel felts. Weight loss was tested by heating the material to 800 °C in a nitrogen atmosphere. During heating from 30 °C to 100 °C, the mass loss of the SCM composite aerogel materials is approximately 6–8%, likely due to the evaporation of residual adsorbed water and solvent. When the temperature continues to rise to around 200 °C, the mass loss is between approximately 12–14%, attributed to the condensation reaction of surface -OH and silanol groups that were not fully substituted, producing water which subsequently evaporates. All SCM samples exhibited typical multi-component polymer decomposition characteristics. Their extrapolated initial decomposition temperatures (T_5_%) decreased slightly with decreasing chitosan content (SCM-1: ~94 °C, SCM-2: ~93 °C, SCM-3: ~94 °C). DTG curves showed that the main decomposition peaks for all three samples were located at ~285 °C, corresponding to the breakage of the chitosan backbone, but the peak height increased with decreasing chitosan content, indicating that the decomposition process was more concentrated. The behavior of SCM is distinctly different from that of pure SiO_2_, clearly attributable to the thermal decomposition process of chitosan, the organic component in the composite material. After 420 °C, the mass loss rate of the samples gradually stabilizes. At 800 °C, the residual masses for SCM-1, SCM-2, and SCM-3 are 70%, 63%, and 54%, respectively. This indicates that the high-temperature thermal stability of the aerogel improves with decreasing CS content and increasing MXene content. The char residue rate is not solely determined by the initial inorganic content, but is mainly influenced by the char-forming potential of the organic components and the high-temperature evolution of MXene. First, as a char-forming polymer, the pyrolysis of CS significantly contributes to the char residue. SCM-1 has the highest CS content, enabling it to form a larger amount of thermally stable carbon during pyrolysis. Second, MXene is not completely inert in high-temperature nitrogen; the decomposition of its surface functional groups and possible slow structural changes lead to some mass loss. Therefore, for SCM-3, its lower chitosan content limits the amount of char formation, while the higher MXene content may introduce more thermal decomposition mass loss pathways, resulting in its lowest final char residue rate. This analysis shows that CS is the key component determining the high-temperature char residue rate in the SCM composite system. A higher char residue typically indicates that the material retains more solid framework at high temperatures, which is consistent with the excellent structural stability exhibited by SCM-1 in thermal runaway tests.

**Figure 6:**
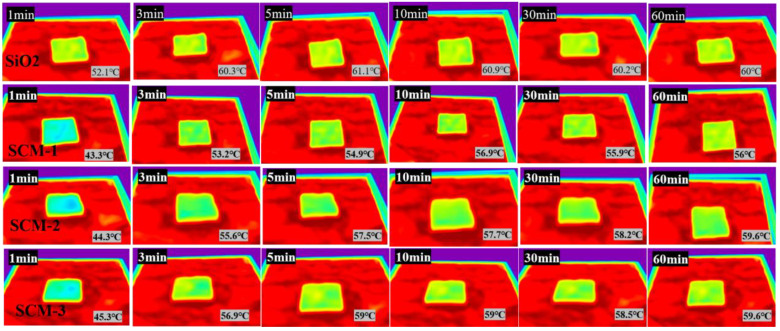
Infrared thermal imaging of composite aerogels with different components.

**Figure 7:**
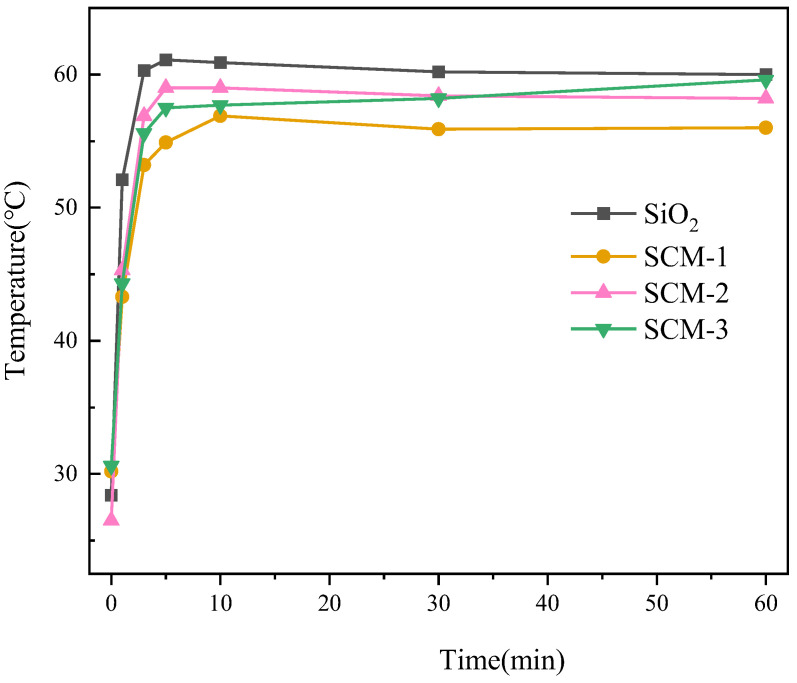
Temperature variation curves of composite aerogel mats with different material ratios under 100 °C heating environment.

The high-temperature insulation performance of SCM was experimentally investigated using a custom-built heating apparatus. A 6 mm-thick sample was placed on a heating stage equipped with an alcohol lamp. When the temperature of the heating stage stabilized, the sample was placed on it for 10 min, and the surface temperature was recorded by an infrared camera, as shown in Figure 6. The temperature of the heating stage was approximately 100 °C. Figure 7 shows the temperature variation curves of the flame-retardant aerogel felts with different material ratios in a 100 °C environment. It can be observed that after heating for 60 min at 100 °C, the surface temperatures of SCM-1, SCM-2, SCM-3, and the SiO_2_ aerogel felt are 56 °C, 58.4 °C, 59.5 °C, and 60 °C, respectively. The surface temperature of SCM-1 is significantly lower than that of the SiO_2_ aerogel felt. This is because MXene has a higher extinction coefficient. Typically, the total thermal conductivity of a composite aerogel can be expressed as the sum of contributions from three components: conduction through the solid, conduction through the gas, and radiative heat transfer. According to Kirchhoff’s law, absorptivity equals emissivity, and the photon thermal conductivity is proportional to temperature and inversely proportional to emissivity. Uniformly distributed MXene nanosheets act as highly efficient infrared shielding agents, significantly reducing the material’s contribution to radiative heat transfer at high temperatures. Therefore, the addition of MXene reduces the photon thermal conductivity of the aerogel material, thereby enhancing its insulation performance in high-temperature environments. This indicates that SCM-1 possesses superior high-temperature insulation properties. Although radiative heat transfer in porous materials is often neglected at room temperature, it increases significantly and becomes a dominant factor at high temperatures. Subsequently, the SCM composite aerogel felt exposed to high temperature still maintains a highly regular layered structure; the nanofibers remain interwoven with each other, and the surface structure shows no collapse of the aerogel framework, reflecting the structural stability of the SCM aerogel felt at high temperatures. The thermal conductivity of SCM-1 measured by the hot disk method at 400 °C is as low as 0.053 W/(m·K), which is much lower than that of the SiO_2_ aerogel felt under the same temperature conditions.

**Figure 8:**
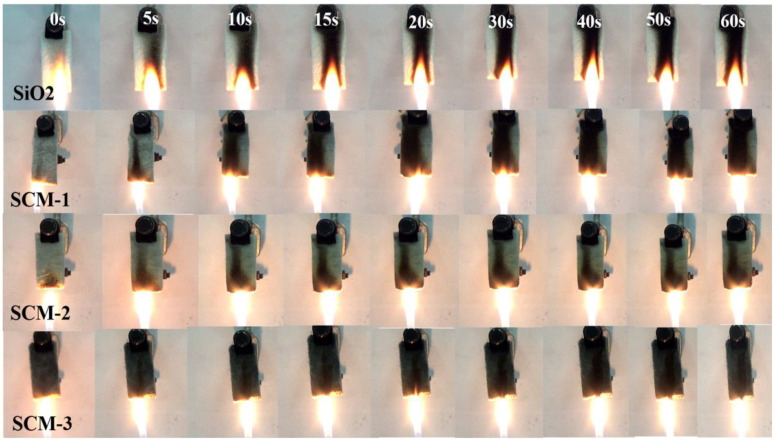
Image of the combustion process of aerogel mat with different components.

The flame retardancy and thermal stability of the SCM composite aerogel felt were evaluated. As shown in Figure 8, after burning for 60 s on an alcohol lamp, the macroscopic shape of the sample remains well-preserved, retaining its initial form without shrinkage or collapse.

**Figure 9:**
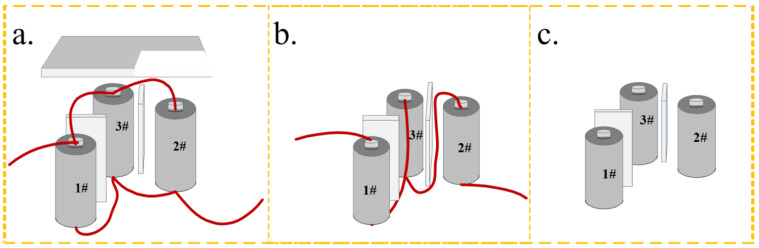
Experimental condition setting: (**a**) Test.1, (**b**) Test.2, (**c**) Test.3.

This study investigates the impact of an SCM insulation layer on the propagation of thermal runaway. The SCM-1 material, with a SiO_2_:CS:MXene solution mass ratio of 1:3:1, was selected as the insulation material. The SCM-1 was cut into pieces measuring 65 mm (length) × 26 mm (width) × 3 mm (thickness) and placed between individual battery cells to act as thermal insulation. To evaluate the ability of SCM composite aerogel mat to suppress thermal runaway propagation, this study constructed a module containing three lithium-ion batteries. The individual cells were commercially available ternary cylindrical 26650 batteries (Sanyo, Osaka, Japan), with a nominal capacity of 5000 mAh and a nominal voltage of 3.6 V. Before the experiment, all batteries were charged and discharged to 100% state of charge (SOC) at room temperature using a battery cycler (NEWARE CT4000T-10V20A, NEWARE, Shenzhen, China), with a charging cutoff voltage of 4.2 V and a discharging cutoff voltage of 2.75 V. The thermal runaway triggering and monitoring system is shown in Figure 9a–c, these three configurations comprised different electrical connections, namely parallel connection, series connection, and no electrical connection. A cylindrical heating rod of the same size as the battery (diameter 26 mm, height 65 mm) was embedded in the pre-reserved gap between the batteries to heat the target battery and trigger thermal runaway. The heating power was set to 200 W, and the maximum heating temperature was set to 400 °C. Type K thermocouples (temperature range 0~1000 °C, accuracy ±1.5 °C) were used to monitor the surface temperature of the batteries. The thermocouples were firmly attached to the geometric center of each battery surface using high-temperature tape. Simultaneously, a data acquisition system was used to record the terminal voltage of each battery.

**Figure 10:**
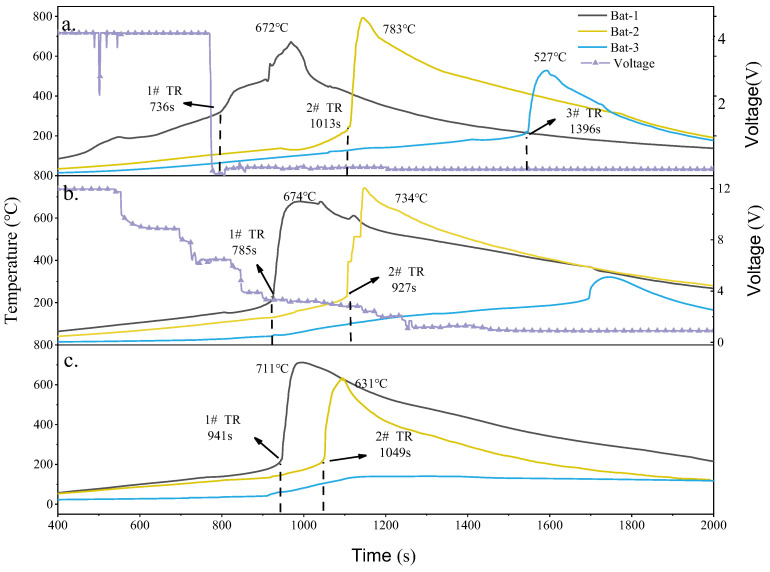
Surface temperature and voltage curves: Aerogel felt placed between cells: (**a**) battery pack in parallel, (**b**) battery pack in series, (**c**) no electrical connection.

Figure 10 shows the surface temperature and voltage variation curves of different electrical systems when using SCM-1 as the insulation material. It can be observed that when thermal runaway occurs, the temperatures of Bat-1 and Bat-2 rise sharply, but only Bat-3 in the parallel module undergoes thermal runaway, as shown in Figure 10a. The results also indicate that the parallel battery system experienced thermal runaway earlier. When SCM-1 was used as the insulation material, the thermal runaway times for the battery modules in Test.1 to Test.3 were 7336 s, 785 s, and 941 s, respectively. The thermal runaway propagation interval from Bat-2 to Bat-3 in the parallel battery system was 283 s, which is 35 s slower compared to using SiO_2_ as the barrier. In Test.2 and Test.3, Bat-3 did not experience thermal runaway. Related research has found that the thermal conductivity of the insulation material has a more significant impact on the thermal runaway propagation barrier. Compared to using SiO_2_ as the barrier, when Bat-1 and Bat-2 near the heating rod were heated, the temperature of Bat-3 showed only a slight increasing trend. Even when Bat-1 and Bat-2 reached their peak temperatures during thermal runaway, Bat-3 was not significantly affected. This is attributed to the MXene component in SCM-1 acting as an infrared opacifier, reducing the photon thermal conductivity of the aerogel material and thereby enhancing its insulation performance under high-temperature conditions. In the parallel battery system, the maximum temperatures of Bat-1, Bat-2, and Bat-3 were 672 °C, 783 °C, and 527 °C, respectively. Compared to using the SiO_2_ aerogel felt as the barrier, the maximum temperatures during thermal runaway were higher. This is because the excellent thermal insulation properties of SCM-1 prevent rapid heat dissipation during the early heating phase of the battery module, leading to heat accumulation. When thermal runaway occurs, the energy generated within the confined space is greater, resulting in a more intense explosion. In the Test.2 and Test.3 battery modules, although Bat-3 did not experience thermal runaway, the maximum temperatures during thermal runaway still reached 734 °C and 711 °C, respectively, both higher than those in battery modules using traditional SiO_2_ aerogel felt as the insulation board under the same electrical connections. It is worth mentioning that, despite this, due to the lower high-temperature thermal conductivity and better thermal stability of the SCM-1 barrier material, thermal runaway propagation was prevented in both the series and no-electrical-connection configurations. The maximum temperature of Bat-3 during thermal runaway in Test.1 was also significantly lower than in previous configurations. In Test.1, within the parallel battery system, Bat-3 discharges to Bat-1 and Bat-2 at a higher rate due to uneven battery operation. Consequently, when thermal runaway occurs, the SOC of Bat-3 is significantly lower than that of the other cells, resulting in its lower temperature rise. This indicates that the excellent high-temperature stability of SCM-1 holds great application potential for inhibiting the propagation of battery thermal runaway.

**Figure 11:**
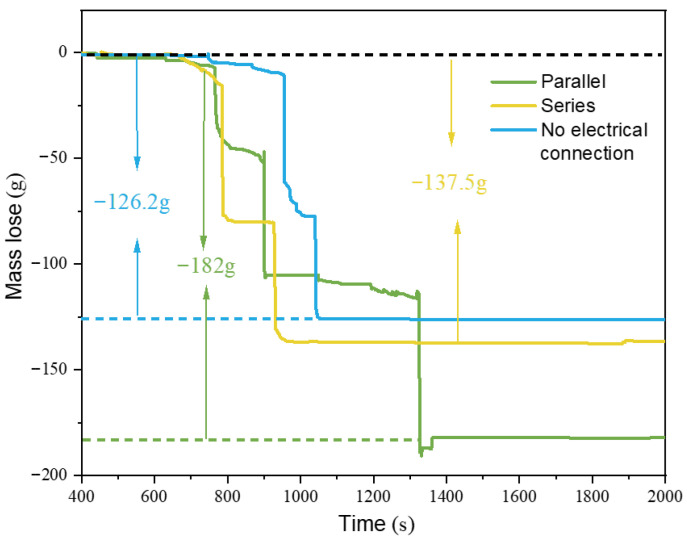
Changes in the quality of battery modules under different operating conditions.

Figure 11 shows the overall mass loss of the battery modules under various configurations when the SCM composite aerogel material was used as the barrier. It can be observed that, except for Test.1, the mass loss curves for the other battery modules where thermal runaway propagation occurred all experienced two sudden drops. After thermal runaway ended, the battery mass slowly stabilized following the loss. It can be seen that when SCM-1 was placed as a barrier in the middle of the battery module, the mass losses for the parallel, series, and no-electrical-connection battery systems were 182 g, 137.5 g, and 126.2 g, respectively. The parallel battery system endured more thermal pathways, thus resulting in greater mass loss. The presence of the top barrier, while reducing the mass loss of Bat-1, led to poorer heat dissipation and heat accumulation, causing Bat-2 and Bat-3 to undergo more severe thermal runaway reactions where active materials were ejected from the battery cells, thus resulting in the most severe mass loss. Therefore, although SCM-1 has better insulation properties and suppressed thermal runaway propagation in both series and no-electrical-connection systems, it also led to poorer heat dissipation performance, more heat accumulation, and higher overall mass loss in the battery system.

**Figure 12:**
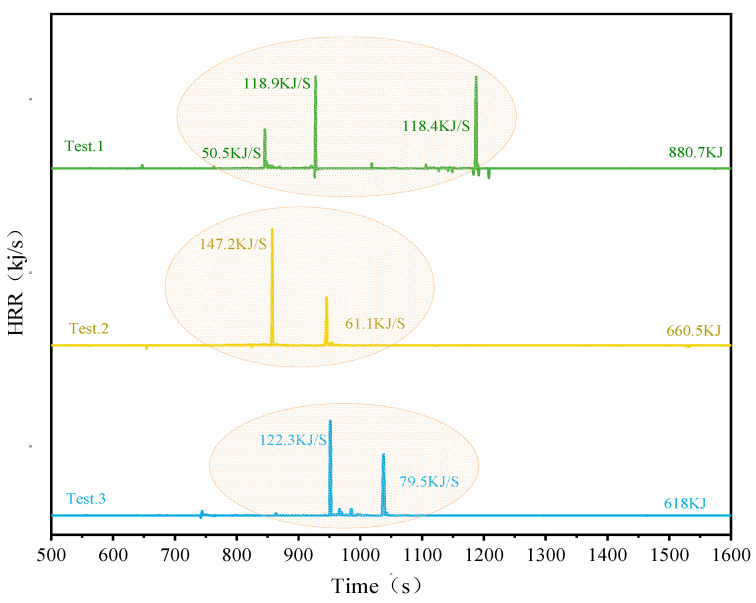
HRR and T HR for all operating conditions.

The Heat Release Rate (HRR) and Total Heat Release (THR) for the three different configurations using SCM-1 as the barrier were calculated. As shown in Figure 12, the peak HRR for the parallel battery pack was 118.9 kJ/s, with a THR of 880.7 kJ. The series module had a peak HRR of 147.2 kJ/s and a THR of 660.5 kJ. The no-electrical-connection battery module had a maximum HRR of 122.3 kJ/s and a THR of 618 kJ. Experimental results reveal key details regarding the use of high-performance aerogel (SCM-1) to suppress heat radiation propagation. While placing SCM-1 between cells (Tests 2 and 3) successfully blocked heat radiation propagation due to its excellent thermal insulation properties, placing it on top of the module (Test.1) resulted in increased internal module temperature and a higher THR than in other tests. This seemingly contradictory phenomenon stems from the dual effect of heat flow within the enclosed module: lateral (inter-cell heat transfer) and longitudinal (cell flame radiation). SCM-1 is highly effective at blocking lateral heat flow that leads to heat propagation. However, when placed on top, it also severely obstructs the necessary vertical heat dissipation path, causing heat to be fed back into the cell module. In Test.1, this resulted in intense heat and flame from the thermally runaway cell being trapped and reflected within the semi-enclosed structure. This accumulation exacerbated the thermal runaway process in the adjacent cell (Bat-3), leading to a more intense heat release reaction and a higher total heat release rate, even though Bat-1 and Bat-2 may have lower heat release rates. Conversely, in tests 2 and 3, the SCM-1 barrier between cells blocked the lateral chain reaction while reducing obstruction to the top surface, allowing some heat to escape and thus preventing catastrophic internal heat buildup. Furthermore, the electrical connection method also affects the results. Compared to the unconnected system, the connected system had slightly higher HRR and THR, which may be due to the additional electrical discharge and ohmic heating generated through the connection lines during thermal runaway. This analysis yields an important practical guideline: SCM-1 aerogel felt demonstrates excellent potential for preventing the propagation of thermal runaway when used as an inter-cell barrier. Therefore, for practical module designs, it is recommended to strategically place SCM-1 insulation material between cells to effectively isolate thermal runaway reactions, thereby improving safety without unintentionally increasing the overall thermal risk due to thermal limitation.

## 3. Conclusions

This study designed and synthesized a novel SiO_2_/Chitosan/MXene (SCM) composite aerogel felt, demonstrating a successful strategy for balancing mechanical strength with exceptional thermal insulation. The incorporation of MXene as an infrared opacifier and chitosan as a reinforcing biopolymer created a synergistic network. This resulted in a material (SCM-1) with a low thermal conductivity of 0.034 W/(m·K) at room temperature and 0.053 W/(m·K) at 400 °C, coupled with a compressive strength of 1.172 MPa, representing a 27% improvement over pure SiO_2_ felt.

The evaluation of SCM-1 within lithium-ion battery modules yielded critical, nuanced insights for thermal safety design. When applied as an inter-cell barrier, SCM-1’s superior insulation effectively blocked thermal runaway propagation, fulfilling its primary design objective. A key finding is that the same insulating property presents a dual effect. When SCM-1 was placed on top of the module, it impeded vertical heat dissipation, leading to significant internal heat accumulation. This exacerbated the fire radiation intensity of the primary thermal runaway event and resulted in thermal runaway propagation with a higher THR. Therefore, SCM aerogel felt is most effective and recommended for use specifically as an inter-cell spacer to isolate failing units, rather than as a full-coverage top blanket.

## 4. Materials and Methods

The SCM aerogel felt materials used in this study were synthesized in the laboratory. The materials employed include tetraethyl orthosilicate (TEOS), chitosan (CS), glutaraldehyde (50%), hydrochloric acid, absolute ethanol (EtOH), glacial acetic acid, hydrochloric acid (HCl), lithium fluoride (LiF), and glass fiber felt. In this experiment, a freeze-drying method was adopted. Based on SiO_2_ aerogel, MXene was introduced as an infrared opacifier, and chitosan was added to enhance the mechanical properties and high-temperature insulation performance of the aerogel felt. On one hand, the SiO_2_ aerogel matrix possesses an ultra-low intrinsic thermal conductivity; on the other hand, MXene serves as an infrared opacifier, helping to reduce thermal radiation. Inspired by the hierarchical characteristics of biological tissues such as honeycombs in nature, the unique network structure and high porosity of chitosan was utilized to bind particles such as silica and further promote the formation of cluster structures. These three components work together to maintain the thermal insulation performance of the aerogel and improve its mechanical properties. Studies have shown that the synthesized composite aerogel achieves a balance between good mechanical strength and thermal insulation performance.

The specific synthesis process was as follows: First, 3.2 g of LiF was weighed and added to a reaction vessel, followed by 40 mL of 9 mol/L hydrochloric acid. The mixture was maintained in a constant-temperature water bath at 40 °C with stirring speed adjusted between 500 and 800 rpm. After stirring for 15 min to ensure sufficient reaction, 2.0 g of Ti_3_AlC_2_ (MAX phase) was added gradually in small portions (approximately 0.1–0.2 g each time), with each subsequent addition only after complete disappearance of bubbles. The reaction proceeded for 48 h. Subsequently, the mixture was centrifuged at 3500 rpm for 1 min. After one round of centrifugation, the precipitate was washed and centrifuged 2–3 times with prepared 2 mol/L dilute hydrochloric acid to remove unreacted LiF. This was followed by repeated washing and centrifugation with deionized water until the supernatant reached a neutral pH (pH ≈ 7). Finally, the precipitate was dried in an oven at 100 °C to obtain multilayer MXene powder.

**Figure 13:**
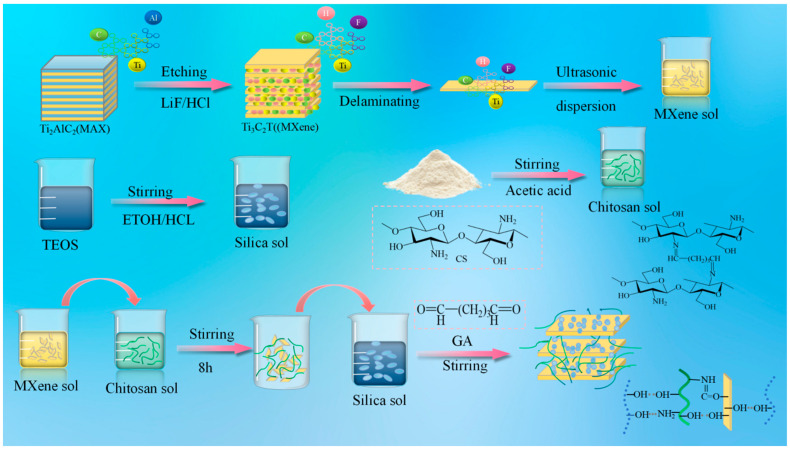
Schematic diagram of the preparation process of SCM composite aerogel material.

For the silica sol preparation, TEOS and ethanol were added to a reaction vessel, followed by dropwise addition of a certain amount of hydrochloric acid and deionized water. The mixture was stirred thoroughly to form a silica sol. Separately, a certain amount of chitosan was added to deionized water to prepare a 1 wt% chitosan solution, to which a specific amount of glacial acetic acid was added dropwise under stirring to obtain a homogeneous chitosan solution. The 1 wt% MXene solution was subjected to ultrasonic dispersion in a water bath sonicator for 60 min to form an MXene aqueous solution. The obtained chitosan solution and MXene aqueous solution were mixed and stirred for 8 h. A glutaraldehyde solvent (with a content equivalent to 1 wt% of chitosan) was added dropwise, and the mixture was vigorously stirred to ensure complete reaction, resulting in a composite sol. The silica precursor and composite sol were mixed in specific ratios. Under vacuum conditions, the glass fiber felt substrate was impregnated into the sol, followed by pre-freezing at low temperature. After pre-freezing, the sample was placed in a freeze-dryer (condenser temperature ≤ −50 °C, chamber pressure ≤ 1 Pa) for freeze-drying for 96 h. The SCM-1, SCM-2, and SCM-3 aerogels correspond to mass ratios of SiO_2_:CS:MXene solution of 1:3:1, 1:2:2, and 3:1:3, respectively, as illustrated in Figure 13.

## Data Availability

The data presented in this study are openly available in article.
